# The screen and the bedside

**DOI:** 10.1017/S1478951526103125

**Published:** 2026-06-29

**Authors:** Vangipuram Sai Shreya

**Affiliations:** Department of General Medicine, Sri Ramachandra Institute of Higher Education and Research, Porur, Chennai, TN, India

The notification arrived at 2:17 a.m. – not about a crashing patient or a critical laboratory result, but about a conversation that had gone quiet.

“Pain is worse tonight. Can’t sleep. The group chat is quiet.”

I read the message twice in the half-dark of the residents’ call room. It came from a patient I had been caring for during my oncology rotation, a man in his thirties with metastatic pancreatic cancer whose world had gradually narrowed to hospital rooms, clinic visits, and the uncertain hope offered by an experimental therapy. I adjusted his overnight pain medications, documented the change, and put my phone down. Yet the message lingered long after the order was signed.

At the time, I assumed he was describing a lack of replies.

Months later, I would wonder whether he had been describing something else entirely.

When I first met him, he seemed to live in two worlds at once. One was the familiar world of medicine, measured in scan reports, infusion schedules, blood tests, and clinic appointments. The other existed almost entirely behind a screen.

He carried a laptop bag everywhere. During admissions it sat beside his bed. During chemotherapy it rested on his knees. While other patients watched television or slept through long afternoons, he moved effortlessly between discussion forums, virtual support groups, and private message threads populated by people scattered across different cities and countries.

Many of them had cancers similar to his. Some were receiving the same treatment. Together they exchanged updates, interpreted scan reports, compared side effects, and tracked one another’s progress with remarkable attention.

One afternoon he showed me a message from a friend he had never met.

“He checks on me more than some of my relatives do,” he said, laughing.

Then, after a pause, he added, “Some of them know me better than people I’ve known my whole life.”

At the time, I found that comforting. Serious illness often isolates people from the rhythms of ordinary life, and his online community seemed to protect him from that isolation. Wherever he was, people noticed when he disappeared for a few days and understood what it meant to wait for results or wake up wondering whether a new pain meant progression.

As the months passed, however, his disease continued to advance.

The changes arrived gradually. He walked more slowly, ate less, and lost weight. Then came the jaundice, increasing fatigue, and the visible effort required for tasks that had once been automatic. Some mornings he needed help moving from the bed to the chair near the window.

The notifications never slowed. Friends checked in at all hours, strangers offered encouragement, and replies accumulated within minutes whenever he shared an update. People celebrated stable scans and mourned disappointing ones. When his pain worsened, suggestions appeared almost immediately.

In many ways, he was surrounded.

Yet something about him was changing that none of those exchanges seemed able to capture.

One rainy afternoon I entered his room and found him staring at his phone. The screen glowed against the dim light. Several unread messages sat stacked beneath one another.

“They keep saying, ‘You got this,’” he said quietly.

I glanced toward the screen. The messages were kind. They were exactly what most of us would probably send.

Then, he lifted his arm.

The skin had become deeply jaundiced. Faint bruises overlapped older ones. A failed intravenous line had left a dark patch near his wrist. He held the arm between us for several seconds before speaking again.

“They know every scan. Every lab result. Every treatment.”

His voice softened.

“But they don’t know what it’s like when someone has to help you turn over in bed.”

The room fell silent.

Outside, rain tapped steadily against the window. Somewhere down the corridor, a monitor alarm sounded and stopped. I stood there holding a tablet filled with laboratory values and medication lists, suddenly aware of how little any of it had to offer the moment unfolding in front of me.

Long after I left the room, I found myself thinking about the way he had held out his arm. The bruises, the yellowing skin, and the effort it had taken simply to lift it seemed to contain more than anything written in the chart. His friends had not failed him. They answered messages at impossible hours, remembered details that even clinicians occasionally forgot, and followed his illness with a devotion that was difficult not to admire. Yet what he had shown me that afternoon was something harder to report. The scans, laboratory values, and treatment plans could be shared. What he had shown me could not.

Several days later I stopped by after rounds. There was nothing urgent to discuss. His laptop was closed. The room smelled faintly of coconut oil from his mother’s hair, and rain blurred the city beyond the window.

We sat quietly for a while. Eventually he spoke. “I spent years building connections.”

A small smile crossed his face. “Hundreds of them.”

He looked toward the ceiling. “And somehow this is still the loneliest I’ve ever been.”

I waited for myself to say something helpful. Nothing came. The quiet that followed felt strangely honest. Yet sitting beside him that afternoon, neither of us seemed interested in fixing the conversation.

Instead, we sat listening to the rain. A nurse entered briefly to check medications and then left. His mother adjusted the blanket around his legs while traffic moved steadily beyond the blurred window. Nothing remarkable happened during those minutes, and perhaps that is why I remember them so clearly. For once, neither of us seemed compelled to turn uncertainty into a conversation.

In the weeks that followed, his condition worsened. Some days he was too tired to speak for long. Other days he seemed determined to hold on to ordinary routines. He continued checking messages and responding when he could.

One evening he played several voice messages from friends he had met online.

Their voices filled the room. One sounded optimistic, another frightened, and a third eventually admitted that he no longer knew what to say.

When the recording ended, Alex laughed softly. “That was the best one.”

I asked him why. “Because he stopped pretending he could fix it.”

The room settled briefly into quiet.

His mother sat in the corner listening. She did not understand the platforms through which these friendships existed, but she listened carefully all the same. The voices had traveled thousands of miles to reach the room, and for a few minutes they occupied the same space as the IV pump, the hospital bed, and the woman who had barely left his side for weeks.

The screen and the bedside existed together. Neither replaced the other.

As his illness progressed, I found myself spending more time in his room without a specific purpose. Sometimes we talked. Sometimes we did not. The conversations became less focused on treatment and more focused on ordinary things: films he enjoyed, places he wanted to revisit, memories from before his diagnosis. Occasionally, he spoke about fear. More often he spoke around it.

Alex died early one morning with his mother holding his hand.

Months later, I still think about the message that arrived at 2:17 a.m. At the time, I thought he was talking about replies. Now I think he was trying to name something harder.

The people in that group chat cared about him deeply. His family cared about him. So did the nurses, physicians, and countless others who moved through that room. Yet there remained a distance none of us could entirely cross, a place where suffering resisted translation.

What I remember now is a room illuminated by two kinds of light. There was the glow of the phone screen resting beside his bed, carrying voices from distant cities and different time zones. And there was the quieter presence of the bedside itself: his mother asleep in a chair, a nurse adjusting a blanket, the ordinary rituals of care unfolding a few feet away.

Neither could fully enter the place he was trying to describe when he lifted his arm and said, “They don’t know what it’s like when someone has to help you turn over in bed.”

Looking back, I think that was what he had been telling me all along. Not that the group chat was quiet, but that some parts of suffering remain beyond the reach of language. What mattered was that the voices kept coming through the screen, and that someone remained at the bedside to hear them.

